# Transient New-Onset Atrial Fibrillation During Acute Chest Pain Unmasking Severe Multivessel and Left Main Coronary Artery Disease: A Case Report

**DOI:** 10.7759/cureus.110656

**Published:** 2026-06-11

**Authors:** Abdulmohsen H Al Mefarrej, Amjaad Almarjan, Fayez A Al Zubair

**Affiliations:** 1 Heart Health Center, King Saud Medical City, Riyadh First Health Cluster, Riyadh, SAU

**Keywords:** acs (acute coronary syndrome), acute non-st elevation myocardial infarction, atrial fibrillation and cad, multivessel coronary artery disease (mvcad), new onset atrial fibrillation

## Abstract

We report a case involving a rare finding observed during a medical evaluation in a patient who visited the emergency department due to acute cardiac chest pain. The patient presented with transient atrial fibrillation (AF) during examination at the emergency department, which was a warning sign observed in this patient, implicating a significant ischemic change on electrocardiography during chest pain. This case showed how the patient was evaluated and treated in accordance with new ischemic changes detected during a transient episode of AF, despite the initial absence of high troponin levels and almost normal electrocardiography findings during sinus activity. Notably, AF during acute chest pain is rarely reported in the literature. The presence of AF might be a clinical marker of major coronary artery disease in symptomatic patients. The current case report was supported by coronary artery angiography findings. The patient received full revascularization treatment, and he was eventually discharged home without recurrence of AF during follow-up. AF played a major diagnostic role in the identification of the patient’s condition.

## Introduction

Atrial fibrillation (AF) is a common complication of acute myocardial infarction (AMI), occurring in approximately 10-21% of patients [1]. AF most commonly develops within the first 24 h after infarction, and it is usually transient in nature [1]. Although it is often attributed to left ventricular failure or left atrial dilation, a distinct and critical mechanism is responsible for direct ischemic injury to the atrial myocardium itself [2]. This atrial infarction can precipitate transient bursts of arrhythmia even in the absence of overt heart failure. Even though AF is commonly recognized as a complication of acute coronary syndrome (ACS), it may occasionally present earlier in the disease course and provide a clue to the presence of severe coronary artery disease. In the former, AF develops secondary to ventricular dysfunction, atrial stretch, inflammation, or hemodynamic deterioration following myocardial injury. While the case suggested a mechanism, transient new-onset AF may arise during active ischemia due to impaired perfusion of the atrial myocardium or specialized conduction tissue, particularly in the setting of extensive proximal coronary disease. Unlike AF occurring as a complication of established infarction, this presentation may precede recognition of the underlying coronary anatomy and therefore function as an early clinical warning sign rather than merely a consequence of ACS.

The hemodynamic sequelae of new-onset AF are profound. Rapid ventricular response combined with loss of atrial systole (atrial kick) can significantly reduce cardiac output, which then promotes cardiogenic shock and further exacerbates coronary ischemia [3]. However, the prognostic significance of transient AF is frequently underestimated. Recent studies have shown that even brief episodes of AF in the setting of acute coronary syndrome are associated with increased in-hospital mortality rates and can be an early clinical marker of extensive coronary artery disease involving the proximal right coronary artery (RCA) or circumflex artery, which supplies the atrial myocardium [4,5].

Herein, we present a case of transient AF acting as an unmasking sign of high-risk coronary artery disease. In this case, the alternating nature of the rhythm oscillating between sinus rhythm and AF was considered as the primary indicator of critical underlying ischemia, thereby emphasizing the need for aggressive angiographic evaluation in such cases.

## Case presentation

Case history and physical examination findings

A 55-year-old male patient from Southeast Asia, not known to have chronic medical illness, started to have exertion-related chest pain for the past 7 months and was controlled with anti-ischemic medications for 6 months before presentation. On top of anti-ischemic medication use, the patient presented to the emergency department with continuous, acute, debilitating chest pain that had started approximately two hours before presentation. The pain was diffuse, compressive in nature, associated with diaphoresis, and progressively increased in intensity. The patient denied any history of syncope, shortness of breath, cough, or fever.

Approximately seven months before presentation, the patient experienced an episode of exertion-related angina lasting about five minutes, which may have represented an early manifestation of the underlying condition. One month later, the symptoms recurred, prompting the patient to seek medical attention. At that time, anti-ischemic therapy was initiated, including aspirin, atorvastatin 80 mg, and isosorbide dinitrate, resulting in adequate symptom control until the current presentation. The patient was loaded with antiplatelet therapy and started blood pressure and pain control medications. The patient had no known chronic medical illnesses and was not taking any long-term medications prior to the initiation of anti-ischemic therapy. His surgical history was significant for cholecystectomy performed 10 years earlier. He had no known drug or food allergies. There was no history of alcohol consumption or illicit drug use. The patient also denied any significant decline in physical activity or energy level compared with his baseline functional status prior to relocating from his home country one year earlier. Regarding family history, his father died between the ages of 85 and 90 years, while his mother passed away at the age of 75 years due to multiple co-morbidities complicated by multiorgan failure. His older brother, who is one year senior to him, is alive and reportedly healthy.

On assessment, the patient continued to experience active chest pain. He was conscious, alert, oriented, and of average body build. His vital signs revealed a blood pressure of 149/88 mmHg, heart rate ranging from 115 to 120 beats/min, oxygen saturation of 98% on room air, and he was not in respiratory distress. Physical examination demonstrated an irregular pulse with normal jugular venous pressure. Cardiac examination revealed audible heart sounds without murmurs or additional sounds. There was no evidence of lower-limb edema. Chest auscultation demonstrated clear bilateral breath sounds. The remainder of the systemic examination was unremarkable. 

Results of pathologic investigations

The patient underwent multiple diagnostic evaluations in the emergency department. Electrocardiography (ECG) performed upon arrival, shown in Figure 1, demonstrated the initial electrical findings. A repeat ECG was obtained after adequate pain control during the emergency department assessment, shown in Figure 2, demonstrating interval changes compared with the initial tracing. Aside from elevated troponin and lactate levels during admission, the remaining laboratory investigations were unremarkable. A summary of the biochemical results is provided in Table 1 and Table 2, and the serial troponin and lactate trends are illustrated in Figure 3.

**Figure 1:**
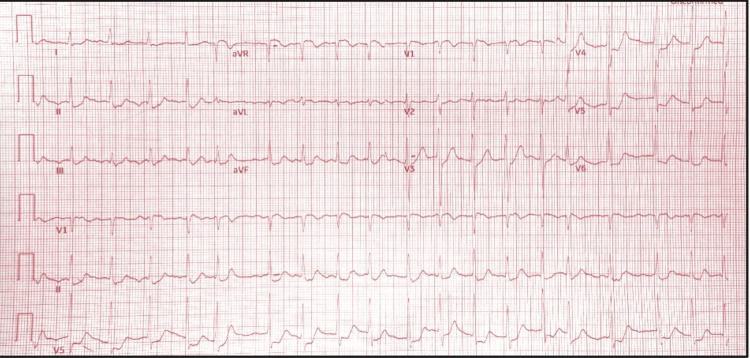
Electrocardiography result during chest pain and atrial fibrillation The irregular rhythm was consistent with atrial fibrillation (AF) and/or occasional premature atrial complexes. A normal axis was observed, and R-wave progression was within normal limits. ST depression was widespread, with ST elevation observed in leads aVR and V1, which correlates with left main coronary artery (LMCA) insufficiency or severe triple-vessel disease.

**Figure 2:**
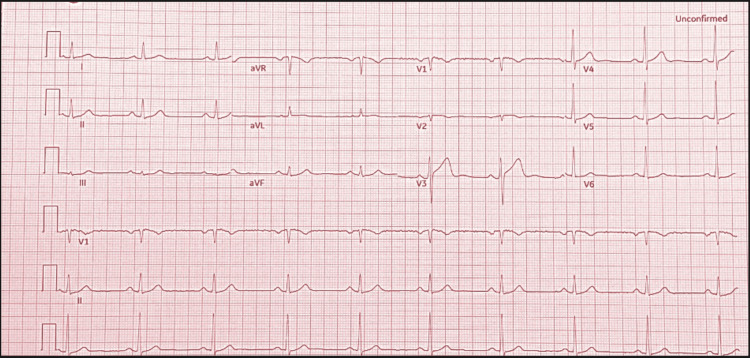
Electrocardiography findings after the initial management The results showed a normal sinus rhythm. There were no ST/T changes indicating acute ischemia.

**Table 1:** Essential laboratory findings LDL, low-density lipoprotein cholesterol; HbA1C, hemoglobin A1C, measuring accumulated blood sugar; TSH, thyroid-stimulating hormone

Lab Type	Reference Range Interpretation		Result
Total Cholesterol	Desirable	0 − 5.18 mmol/	4.01
	Borderline	5.18 - 6.19 mmol/L	
	High	≥ 6.22 mmol/L	
LDL	Optimal	< 2.59 mmol/L	2.96
	Near optimal	2.59 - 3.34 mmol/L	
	Borderline high	3.37 - 4.12 mmol/L	
	High	4.14 - 4.89mmol/L	
	Very high	≥ 4.92 mmol/L	
HbA1C	Normal	<5.7 %	5.50%
	Pre-diabetes	5.7% - 6.4%	
	Diabetes	≥ 6.5%	
TSH	Normal	0.53 - 4.94 mIU/L	1.35
Free T4	Normal	9.01 - 19.05 pmol/L	11.7

**Table 2:** Laboratory examination findings obtained on the day of admission and discharge WBC, white blood cell; HGB, hemoglobin

Lab Type	Reference Range	Admission Results	Results At Discharge
Creatinine	63.6 - 110.5 umol/L	66.8	72
Potassium	3.5 - 5.1 mmol/L	4.5	4.4
Sodium	136 - 145 mmol/L	137	139
WBC	4 - 11 (10*3/uL)	7.35	7.72
HGB	13.5 - 18.0 g/dL	16.7	14.8

**Figure 3:**
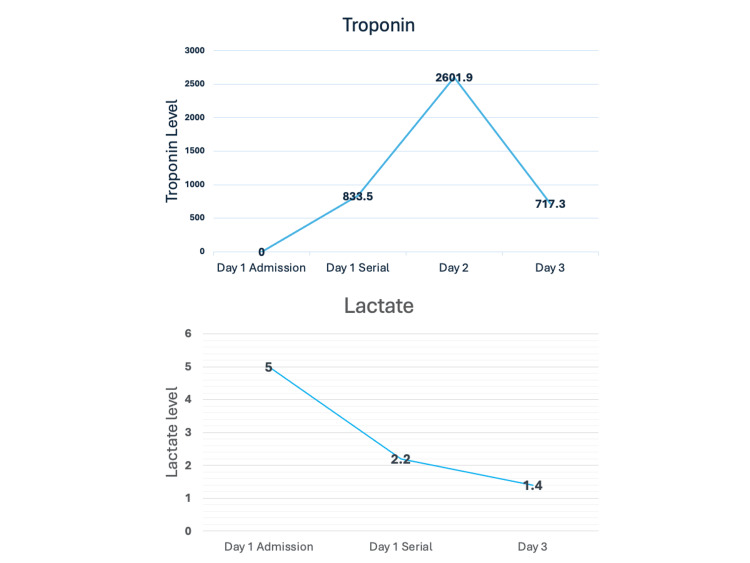
Representative day-wise trends of troponin and lactate levels The timeline recordings for troponin levels are shown in the upper image, and those for lactate levels in the lower image. Reference Range: Lactate (0.9 - 1.7 mmol/L); Troponin (0.0 - 34.2 ng/L)

The patient's echocardiography exhibited normal left ventricular (LV) function, and mild concentric LV hypertrophy was observed, with an estimated ejection fraction of 55%. The LV wall motion, gross size of the right ventricle, right ventricular systolic function, and left atrial size were normal. The atrial septum had an aneurysm. Trace mitral regurgitation and mild tricuspid regurgitation were noted. The right ventricular systolic pressure was normal. There was no hemodynamically significant valvular aortic stenosis. The pericardium appeared normal, and pericardial effusion was not detected, as shown in Figure 4.

**Figure 4:**
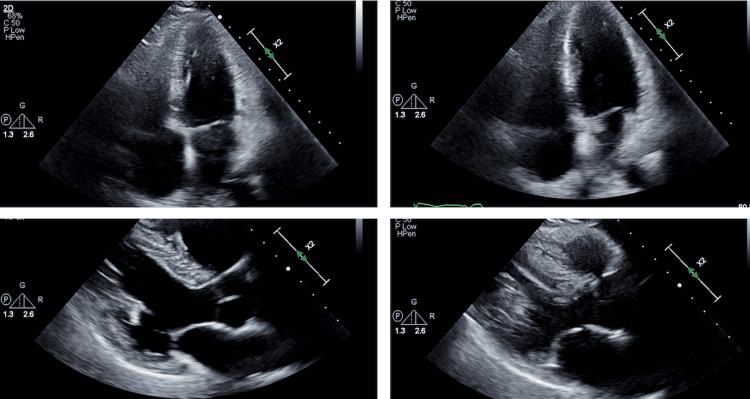
Multiple views comparing systole and diastole on echocardiography Top left image: View of the four chambers during systole. Top right image: View of the four chambers during diastole. Bottom right image: View of the parasternal long axis during systole. Bottom left image: View of the parasternal long axis during diastole, which shows, in general, good left ventricular size and function and no clear regional wall motion abnormality.

Treatment plan and expected and actual outcomes

Despite the initial measurements, the patient still complained of chest pain. During this time, laboratory work was performed, and results were pending (Table 1, Figure 3). The progress of the patient’s condition was shared with the on-call team consultants (interventionist and admitting). With consideration of ongoing ischemic chest pain, the patient was prepared for urgent coronary angiography and coronary intervention, if required. The working diagnosis was acute coronary syndrome (non-ST elevation). The differential diagnosis was acute AF, in addition to type II MI.

The patient underwent coronary angiography, and the results are presented in Figure 5. The patient had a heart team discussion with interventionists, imaging specialists, and cardiac surgeons. The consensus was to offer cardiac surgery as the best treatment strategy. But the patient declined surgery and preferred elective management with PCI himself. He then underwent intravascular ultrasound-guided PCI of the RCA with three drug-eluting stents (34 mm at the mid-distal, 48 mm at the ostial-mid, and 12 mm at the ostial cover lesion). The patient also underwent recatheterization with intravascular ultrasound-guided PCI of the LMCA and left anterior descending (LAD) coronary arteries with two drug-eluting stents (38 mm at the LMCA and 15 mm at the mid-LAD artery), with minimal overlap. Successful PCI outcomes were achieved in two sessions.

**Figure 5:**
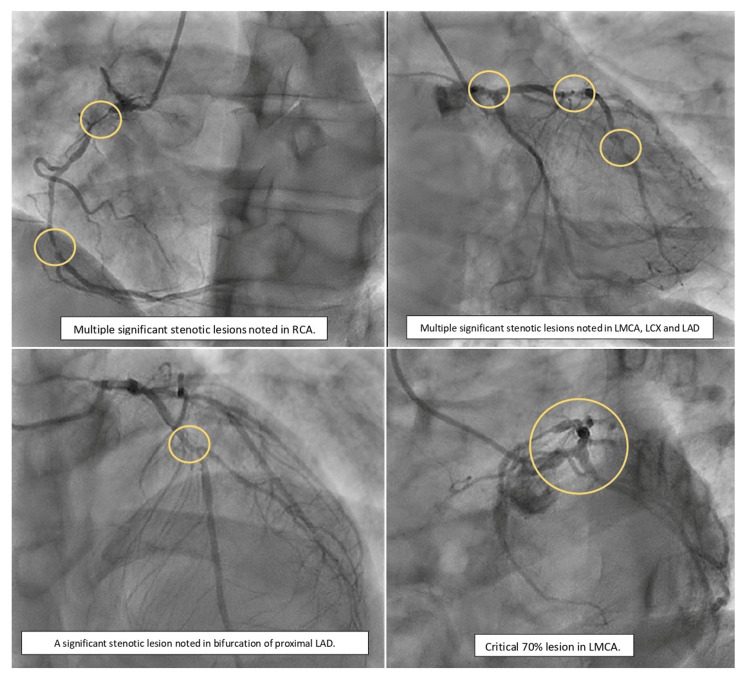
Sample images captured on coronary angiography Left top image: Left anterior oblique caudal view of the right coronary artery. Left bottom image: Right anterior oblique cranial view of the left anterior descending coronary artery. Right top image: Right anterior oblique caudal view of the left coronary artery system. Right bottom image: Left anterior oblique caudal view of the left coronary system. Description of findings: Left main coronary artery: A 70% heavily calcified stenotic lesion was noted in the left main coronary artery (LMCA); Left anterior descending artery: A 90% bifurcation lesion was found in the proximal left anterior descending (LAD) artery; Circumflex artery: A 90% lesion was observed in the distal circumflex (LCX) artery; Right coronary artery (RCA): A 99% lesion was noted in the proximal RCA. A 99% lesion was also found in the middle part of the RCA.

The patient was discharged home in good condition with anti-ischemic medications, including dual antiplatelet therapy, statin, beta-blocker, and angiotensin-converting enzyme inhibitor. The ECG findings upon discharge revealed sinus rhythm. During the one-month follow-up at the outpatient department, the patient was in good clinical condition. He had a stable chest condition and did not complain of chest discomfort. He had no ED visits for the succeeding six months. AF did not recur.

## Discussion

New-onset AF in ACS was seldom reported in the literature. A sub-analysis of a large cohort of acute myocardial infarction cases showed that new-onset AF indicates a poor prognosis, particularly if it persisted > 24 hours. It was shown that transient AF is associated with a higher risk of adverse events than post-coronary artery bypass grafting surgery-related AF, or even a patient with known AF. The risk was studied in primary and secondary outcomes of major adverse cardiovascular events (MACE), stroke, or mortality [5]. It is worth mentioning that this case is a single observation, and causality cannot be definitively proven.

In general, transient electrical AF is defined as a self-limiting episode of arrhythmia, often precipitated by reversible triggers, such as inflammation, metabolic abnormalities, and acute hemodynamic instability [6]. According to recent guidelines, this definition applies even to the first detected episode, even if it resolves spontaneously. The incidence rate of AF after acute myocardial infarction (AMI) is 10-20% within the first 30 days, and AF is typically associated with advanced age, higher Killip classification, and severe LV dysfunction [1]. However, our case is distinct as the patient did not present with severe LV failure, which indicated an alternative pathophysiological driver.

AF after myocardial infarction is traditionally caused by ventricular failure and atrial stretch. However, direct ischemic injury to the atrium is an important, often overlooked mechanism. Autopsy studies have revealed that atrial infarction, predominantly affecting the right atrium, occurs in 7-17% of patients with AMI [7]. The left atrium is frequently spared, likely due to the protective effect of high oxygen tension provided by direct contact with oxygenated blood within the chamber [7].

The onset of AF during AMI is a pivotal clinical event that often serves as a marker of hemodynamic deterioration rather than as a primary electrical disorder. Although traditionally attributed to elevated left ventricle end-diastolic pressure and secondary left atrial stretching [8], this case illustrates a distinct and important mechanism. In particular, direct ischemic injury to the atrial myocardium unmasks high-risk coronary anatomy. The pathophysiology of AF in the setting of acute coronary syndrome is multifactorial. In cases in which the arrhythmia is transient or alternates with sinus rhythm, as observed in this patient, the mechanism is likely driven by transient ischemia of the atrial conduction system. The sinus nodal artery originates from the RCA in approximately 60% of individuals and from the left circumflex artery in 40% [9]. Similarly, the atrioventricular nodal artery arises from the RCA in 90% of the population [10]. Occlusion or severe stenosis proximal to these origins can lead to atrial infarction or ischemia of the specialized conduction tissues, causing immediate electrical instability [11]. Ischemic AF is distinct from heart failure-related AF. Kyriakidis et al. showed that patients with MI at the inferior wall who develop AF almost invariably present with occlusion of the proximal RCA, affecting the blood supply to the right atrium and sinus node [12]. This phenomenon is correlated with our finding that the alternating arrhythmia was not merely a bystander but a specific signpost for severe proximal disease. The alternating nature oscillating between AF and sinus rhythm likely reflects intermittent perfusion and varying vagal tones often observed in inferior or posterior alterations, creating a substrate of re-entry circuits within the stunned atrial myocardium [13].

The presence of new-onset AF, even when transient, is associated with an unfavorable prognosis. A meta-analysis has shown that new-onset AF is associated with an increased risk of in-hospital mortality and major bleeding events compared with patients who remain in sinus rhythm [14]. In particular, the loss of the atrial kick in non-compliant, ischemic ventricles can reduce cardiac output by 20-30%, converting a stable ST-elevation MI/non-ST elevation MI into cardiogenic shock [15]. Therefore, the occurrence of this arrhythmia should act as a stress test, revealing that the myocardium is under significant metabolic strain. Clinicians often dismiss transient AF once sinus rhythm is restored, attributing it to sympathetic surge [16]. However, according to Lazar et al., atrial infarction is often missed on standard 12-lead ECG because the low-voltage atrial depolarization wave is buried within the QRS wave complex [2]. The appearance of AF may be the only external evidence of focal atrial necrosis.

The standard management strategies for AF in AMI prioritize rate control and hemodynamic stabilization. Beta-blockers, such as carvedilol, are effective in reducing myocardial oxygen demand, and digoxin is an alternative in the setting of left ventricular dysfunction [1]. Platia et al. revealed that esmolol achieved conversion to sinus rhythm in 50% of patients with new-onset AF, compared with only 12% in those managed with verapamil [17]. However, our case demonstrated a unique response. In particular, the patient achieved spontaneous conversion to sinus rhythm only after the administration of nitroglycerin. The patient’s hypertension and arrhythmia were refractory to adequate analgesia, implying that the clinical presentation was caused by supply-demand ischemia rather than by pain-induced sympathetic surge. This phenomenon is in accordance with the supply-demand mismatch described by Murphy et al., where obstructive coronary lesions compromise blood flow during periods of stress [18]. This concept was further elaborated by de Lemos et al. in the context of type 2 MI, where diffuse obstructive disease precipitates ischemia in the setting of stressors such as hypertension [19]. In the current case, coronary angiography revealed severe left main coronary artery (LMCA) and three-vessel coronary disease. Although studies on LMCA disease, particularly transient AF, are limited, this case suggests that transient AF can be an unmasking event for important proximal coronary anatomy.

## Conclusions

The current case suggests that transient, paroxysmal AF presenting during acute ischemic chest pain should serve as an important clinical sign that warrants vigilant evaluation for critical underlying proximal coronary artery or multivessel disease.
